# Impact of diabetes distress on glycemic control and diabetic complications in type 2 diabetes mellitus

**DOI:** 10.1038/s41598-024-55901-0

**Published:** 2024-03-06

**Authors:** Hye-Sun Park, Yongin Cho, Da Hea Seo, Seong Hee Ahn, Seongbin Hong, Young Ju Suh, Suk Chon, Jeong-Taek Woo, Sei Hyun Baik, Kwan Woo Lee, So Hun Kim

**Affiliations:** 1 Department of Internal Medicine, Gangnam Severance Hospital, Yonsei University College of Medicine, Seoul, Korea; 2 Department of Endocrinology and Metabolism, Inha University College of Medicine, Incheon, Korea; 3 Department of Biomedical Sciences, Inha University College of Medicine, Incheon, Korea; 4 Department of Endocrinology and Metabolism, Kyung Hee University School of Medicine, Seoul, Korea; 5 Department of Internal Medicine, Korea University College of Medicine, Seoul, Korea; 6 Department of Endocrinology and Metabolism, Ajou University School of Medicine, Suwon, Republic of Korea; 7 Division of Endocrinology and Metabolism, Department of Internal Medicine, Inha University College of Medicine, 27, Inhang-ro, Joong-gu, Incheon, 22332 Korea

**Keywords:** Diabetes complications, Type 2 diabetes

## Abstract

The effect of diabetes distress on glycemic control and its association with diabetes complications is still poorly understood. We aimed to study the clinical features of patients with high diabetes distress, focusing on changes in glycemic control and risk of diabetic complications. From the Korean National Diabetes Program data, we investigated 1862 individuals with type 2 diabetes mellitus (T2DM) who completed diabetic complication studies and the Korean version of the Problem Areas in Diabetes Survey (PAID-K). A total score of PAID-K ≥ 40 was considered indicative of high distress. Individuals with high distress (n = 589) had significantly higher levels of glycated hemoglobin than those without distress (7.4% vs. 7.1%, p < 0.001). This trend persisted throughout the 3-year follow-up period. Higher PAID-K scores were associated with younger age, female gender, longer duration of diabetes, and higher carbohydrate intake (all p < 0.05). There was a significant association between high distress and diabetic neuropathy (adjusted odds ratio, 1.63; p = 0.002), but no significant association was found with other complications, including retinopathy, albuminuria, and carotid artery plaque. In conclusion, high diabetes distress was associated with uncontrolled hyperglycemia and higher odds of having diabetic neuropathy.

## Introduction

Diabetes distress refers to negative emotional or affective experiences that result from psychosocial adjustment to diabetes^1^. This is distinct from emotional stress, such as depression or anxiety because diabetes distress is associated with daily life experience with diabetes, but not a generic feeling^1^. Diabetes distress often arises from concerns about food, future complications, and uncomfortable social interactions^2^. Furthermore, patients with distress are more likely to have a severe diabetes symptom burden, work disability, and higher healthcare costs^3,4^. The attention to diabetic distress has been increasing because it is common in patients with diabetes, which may affect its management. A meta-analysis reported that up to 36% of patients with type 2 diabetes mellitus (T2DM) experience diabetes distress^5^.

It was believed that diabetes distress could be associated with better diabetes care and compliance because concerns about diabetes might motivate patients to follow self-care recommendations^1^. However, it has been reported that diabetes distress is associated with lower levels of self-care and higher glycated hemoglobin (HbA1c) levels, rebutting the previous hypothesis^6^. Concerns about diabetic complications in patients with high diabetes distress have been raised accordingly. Previous studies have shown that depression and anxiety are associated with an increased risk of diabetic complications^7,8^. However, a limited number of studies have assessed the relationship between diabetes distress and diabetic complications^9^. Additionally, the association between diabetes distress and diabetic complication in Koreans has not been well established, although ethnicity is independently associated with high diabetic stress and the responses to stress may vary across countries and ethnicities^10^. In this study, we aimed to study the association between diabetes distress, glycemic control, and diabetic complications and further investigate the clinical features in patients with high diabetes distress using data from the Korean National Diabetes Program (KNDP) cohort.

## Methods

### Study participants

The KNDP cohort database was used for this study. The KNDP is a prospective, multicenter, observational cohort study performed at 13 research hospitals in South Korea, started in May 2006. The study primarily aimed to investigate mortality and diabetes-related complications and included patients with T2DM and those at a high risk of diabetes. Clinical data including medical histories, physical examinations, laboratory tests and surveys were collected. Details of the study have been previously published^11^. We included 1,862 individuals from the KNDP cohort who satisfied the following criteria: (1) diagnosed with T2DM according to the American Diabetes Association criteria^12^; and (2) those who had completed the questionnaire, including a diabetes distress survey conducted during the third year of follow-up after entering the cohort. The baseline characteristics at the time of the distress survey, including age, sex, anthropometrics, smoking and alcohol history, diabetes duration, medications, and laboratory data were collected from the dataset. Among them, we excluded variables with missing data exceeding 25%^13^. For those who completed 3-years follow-up after the survey (928 patients, 49.8%), laboratory data related to glucose control for 3 years were additionally collected. The comprehensive flow of the enrollment process for study participants is represented in Supplementary Fig. 1. All patients with T2DM were managed in accordance with the guidelines of the Korean Diabetes Association. All the participants provided written informed consent, and the study was approved by the Ethics Committee of the Inha University Hospital (2022-02-003).

### Clinical and laboratory measurements

Body mass index (BMI) was calculated as weight (kg) divided by height in meters squared (m^2^). Laboratory data, including glycated hemoglobin (HbA1c), triglyceride, high-density lipoprotein cholesterol (HDL-C), low-density lipoprotein cholesterol (LDL-C), blood urea nitrogen, and creatinine were measured using routine laboratory methods. The estimated glomerular filtration rate (eGFR) was assessed using the Modification of Diet in Renal Disease Study equation (MDRD)^14^. The HbA1c measurements at 3 years after the survey were used to assess prospective glycemic control. A HbA1c level < 6.5% was defined as well controlled diabetes, whereas HbA1c level ≥ 6.5% was defined as uncontrolled diabetes. Trained dietitians interviewed the participants using a 24-h dietary recall questionnaire, as previously described^15^. Daily intakes of total calories, carbohydrates, proteins, total fat, and fiber were evaluated. Energy-adjusted nutrient intake was described as a percentage of total energy for carbohydrate, protein, and fat and g/1000 kcal for dietary fiber.

### Diabetic complications

Data on diabetic complications, including carotid artery plaque, retinopathy, albuminuria, and neuropathy were collected. The presence of carotid artery plaque was evaluated in both common carotid arteries using high-resolution B-mode ultrasonography, as previously described^16^. A localized structure encroaching into the arterial lumen with a thickness > 1.2 mm was defined as carotid plaque^17^. Diabetic retinopathy was diagnosed using fundoscopic examination and confirmed when leaky blood vessels, retinal swelling, retinal exudates, or any changes in retinal blood vessels were observed^18^. Urinary creatinine and albumin levels were measured using a turbidimetric assay (Cobas Integra, Roche Diagnostics, Mannheim, Germany) with random urine samples. Albuminuria was defined as a urinary albumin-creatinine ratio ≥ 30 μg/mg Cr^16^. Diabetic neuropathy was diagnosed when any of the following three criteria were met: (1) symptoms of neuropathy (2) signs of neuropathy and (3) confirmation by a nerve conduction test. Symptoms of neuropathy were assessed by self-report questionnaire using the Diabetic Neuropathy Symptom score^19^. Signs of neuropathy were evaluated using the the Diabetic Neuropathy Examination Score^20^.

### Assessment of diabetes-related emotional distress

The Problem Areas In Diabetes (PAID) assessment was initially designed to measure the emotional burden in patients with diabetes^21^. To make this evaluation applicable to Korean, the PAID evaluation was translated into the Korean version (PAID-K) as previously described^22^. Each item was scored on a five-point Likert scale, ranging between 0–4. The total sum of 20 items was multiplied by 1.25 and yields a final possible score of 0–100, with higher scores indicating higher levels of diabetes-related emotional distress^23^. For the PAID scale, a score of 40 or higher is indicative of high diabetes distress and demonstrates discriminative validity^24,25^. In Asian populations, the same cutoff value of 40 has been consistently used to define high diabetes distress^26–28^. Therefore, in this study, we adopted the same cutoff to define high diabetes distress, which is similar to the highest tertile (score > 38) of the current study population.

### Statistical analysis

The study participants were classified according to their PAID-K score, as low and high distress group and characteristics of these groups were compared. The Shapiro–Wilk test was used to evaluate normality of variables. Continuous variables are expressed as median with interquartile range (IQR). Mann–Whitney U were used for continuous variables analysis. Daily intake of total calories, carbohydrates, proteins, total fat, and fiber was additionally analyzed by Quade’s ANCOVA with sex adjustment. All the categorical variables were expressed as numbers (%) and compared using the χ^2^ analysis. Multiple linear regression models were employed to identify variables which were independently associated with variations in PAID-K score. Univariable regression analysis of the model-developing set was used to select significant determinants (p < 0.05) for the subsequent development of the multivariable linear regression model.

To confirm the effect of high distress on glucose control, a subgroup of patients who completed a 3-year follow-up after the survey (n = 928, 49.8% of the total population) was analyzed. We conducted a χ2 analysis to compare the proportion of patients with well-controlled diabetes, defined as HbA1c < 6.5%, at both baseline and 3 years following the survey. Furthermore, within this subgroup, we utilized multivariable logistic regression to investigate whether high distress independently correlated with inadequate glycemic control. The same analyses was repeated in patients stratified by glycemic control state (HbA1c < 6.5% or HbA1c ≥ 6.5%) at the time of the survey. The odds ratio (OR) of diabetic complications, including neuropathy, retinopathy, albuminuria, and carotid artery plaque was calculated using multivariable logistic regression analysis, and various confounding factors were adjusted in a stepwise manner. Age and sex were adjusted in Model 2; BMI and duration of diabetes were further adjusted in Model 3; systolic blood pressure, HbA1c, and eGFR in Model 4; and use of insulin, statin, and alcohol/smoking consumption in Model 5. Statistical significance was set at a p value of < 0.05. All statistical analyses were performed using IBM SPSS Statistics version 26.0 (IBM Corp., Armonk, NY, USA).

## Results

A total of 1862 participants were enrolled in the study, and the baseline characteristics of the study participants according to the PAID-K score are shown in Table 1. A PAID-K score ≥ 40 was defined as high distress. Of the 1,862 study participants, 589 (31.6%) were classified as high distress group. Participants with high distress were likely to be younger, predominantly women, and have a longer duration of diabetes (all p < 0.001). Regarding laboratory tests, they exhibited higher fasting glucose, HbA1c, and LDL-C and a higher proportion was using insulin therapy compared to those with low distress. (Table 1). For dietary patterns, participants with high distress had a pattern of lower energy intake, higher carbohydrate intake, and reduced fiber intake. However, upon adjusting for sex, only elevated carbohydrate intake and reduced fiber intake remained significant in the high distress group. (Table 1).

**Table 1:** Baseline characteristics of study participants according to the PAID-K score.

Variables	Low distress (N=1273)	High distress (N=589)	p value	p' value
Female, n (%)	535 (42.0)	311 (52.8)	< 0.001	
Age, years	59.0 (53.0–65.0)	56.0 (50.0–64.0)	< 0.001	
Duration of diabetes, years	8.0 (4.0–13.0)	10.0 (5.0–15.0)	< 0.001	
BMI, kg/m^2^	25.0 (23.2–27.0)	25.0 (23.0–27.2)	0.912	
Waist circumference, cm	88.0 (83.0–93.0)	87.0 (81.8–92.0)	0.024	
Systolic BP, mmHg	124.0 (118.0–132.5)	124.0 (118.0–131.0)	0.922	
Diastolic BP, mmHg	78.0 (70.0–81.0)	78.0 (70.0–80.0)	0.726	
Fasting blood glucose, mg/dL	126.0 (110.0–146.0)	133.0 (110.0–152.0)	0.032	
HbA1c, %	6.9 (6.4–7.6)	7.2 (6.6–8.0)	< 0.001	
Triglyceride, mg/dL	117.0 (82.0–168.0)	116.0 (75.0–175.8)	0.382	
HDL-cholesterol, mg/dL	48.0 (41.0–56.0)	49.0 (42.0–58.0)	0.060	
LDL-cholesterol, mg/dL	81.2 (62.0–192.6)	85.3 (66.0–107.9)	0.012	
uACR	12.1 (6.1–29.8)	9.9 (5.7–37.5)	0.281	
eGFR MDRD, mL/min/1.73 m^2^	88.4 (73.9–105.2)	87.9 (74.6–107.1)	0.640	
Alcohol consumption, current. n/total (%)	484/999 (48.4)	235/532 (44.2)	0.110	
Smoking, ever, n/total (%)	435/1005 (43.3)	213/534 (39.9)	0.438	
Statin use, n/total (%)	536/1226 (43.7)	224/571 (39.2)	0.073	
Energy intake, kcal/day	1714.9 (1512.5–1921.2)	1,666.6 (1437.3–1915.0)	0.005	0.271
Carbohydrate intake, % energy	58.5 (53.2–63.7)	59.5 (55.1–64.6)	0.004	0.045
Protein intake, % energy	17.2 (15.6–18.8)	17.2 (15.3–19.0)	0.518	0.479
Fat intake, % energy	22.9 (19.5–27.2)	22.8 (19.4–26.4)	0.338	0.440
Fiber intake, g/1000 kcal	26.4 (21.6–31.4)	25.4 (20.5–30.5)	0.020	0.036
Diabetic medications			0.001	
OADs only	664/1226 (54.2)	291/575 (50.6)		
OADs plus insulin	107/1226 (8.7)	64/575 (11.1)		
Insulin only	67/1226 (5.5)	57/575 (9.9)		
Diabetic complications				
Presence of carotid plaque, n/total (%)	631/1014 (62.2)	275/470 (58.5)	0.172	
Diabetic retinopathy, n/total (%)	251/1017 (24.7)	132/475 (27.8)	0.200	
Albuminuria, n/total (%)	156/722 (21.6)	89/306 (29.1)	0.010	
Diabetic neuropathy, n/total (%)	159/781 (20.4)	135/433 (31.2)	< 0.001	

A score ≥ 40 on the Korean version of the Problem Areas in Diabetes Survey was defined as high distress. Continuous variables are expressed as median with interquartile range (IQR). Mann–Whitney U were used for continuous variables analysis.

*PAID-K* Korean version of the Problem Areas in Diabetes Survey, *BMI* body mass index, *BP* blood pressure, *HbA1c* glycated hemoglobin, *HDL* high-density lipoprotein, *LDL* low-density lipoprotein, *uACR* urinary albumin-creatinine ratio, *eGFR* estimated glomerular filtration rate, *MDRD* modification of diet in renal disease, *OAD* oral antidiabetic drug.

The p’ value is derived from Quade’s ANCOVA with gender adjustment.

The factors associated with PAID-K scores were examined, as shown in Table 2. In univariable analysis, higher PAID-K scores correlated with women, younger age, longer diabetes duration, higher HbA1c, HDL-C, and LDL-C levels. Additionally, higher PAID-K scores were associated with lower total energy intake, higher carbohydrate intake and lower fiber intake. Multivariable linear regression analysis was performed to identify variables which were independently associated with variations in PAID-K score. Age, gender, duration of diabetes, HbA1c, HDL-C, LDL-C, total energy intake, carbohydrate intake and fiber intake were included as independent variables. Among the independent variables included in the multivariable linear regression model, age (β = -0.126, p < 0.001), female gender (β = 0.089, p = 0.006), duration of diabetes (β = 0.119, p < 0.001), and carbohydrate intake (β = 0.083, p = 0.006) were independently associated with PAID-K scores after adjusting for the other co-variables (Table 2).

**Table 2:** Linear regression analysis of the association between PAID-K score and participants’ clinical characteristics and laboratory findings.

	Univariable analysis		Multivariable analysis	
	Standardized coefficients (β)	*p*-value	Standardized coefficients (β)	*p*-value
Female	0.100	< 0.001	0.089	0.006
Age, years	− 0.087	< 0.001	− 0.126	< 0.001
Diabetes duration , years	0.108	< 0.001	0.119	< 0.001
Body weight, kg	− 0.032	0.184		
BMI, kg/m^2^	− 0.006	0.787		
Systolic BP, mmHg	0.005	0.825		
Waist circumference, cm	− 0.051	0.050		
Fasting blood glucose, mg/dL	0.047	0.083		
HbA1c, %	0.106	< 0.001	0.044	0.120
HDL-cholesterol, mg/dL	0.050	0.042	0.022	0.247
Triglyceride, mg/dL	0.021	0.380		
LDL- cholesterol, mg/dL	0.066	0.008	0.079	0.772
Energy intake, kcal/day	− 0.079	0.002	− 0.009	0.815
Protein intake, % energy	− 0.049	0.060		
Carbohydrate intake, % energy	0.084	0.001	0.083	0.006
Fat intake, % energy	− 0.010	0.687		
Fiber intake, g/1000 kcal	− 0.057	0.029	− 0.047	0.142

*PAID-K* Korean version of the Problem Areas in Diabetes Survey, *BMI* body mass index, *BP* blood pressure, *HbA1c* glycated hemoglobin, *HDL* high-density lipoprotein, *LDL* low-density lipoprotein.

The high distress group had a higher prevalence of albuminuria and diabetic neuropathy (29.1% vs. 21.6%; p = 0.010, 31.2% vs. 20.4%; p < 0.001, respectively, Table 1). The association between high distress and diabetic complications was analyzed (Table 3). Patients with high distress had increased odds of diabetic neuropathy (OR: 1.67, 95% confidence interval [CI]: 1.25–2.23), which remained significant after adjusting for multiple variables, including age, sex, BMI, duration of diabetes, systolic blood pressure, HbA1c, eGFR, use of insulin, use of statin, and alcohol intake and smoking (adjusted OR: 1.66, 95% CI: 1.22–2.26). Diabetic retinopathy, albuminuria and carotid artery plaques were not associated with high levels of distress.

**Table 3:** Odds ratio of having diabetic complications in subjects with high distress (≥ 40 PAID-K score).

Total	Neuropathy (N = 991)	p value	Retinopathy (N = 1072)	p value	Albuminuria (N = 729)	p value	Carotid plaque (N = 1129)	p value
Model 1	1.67 (1.25–2.23)	0.001	1.31 (0.98–1.74)	0.066	1.38 (0.98–1.95)	0.063	0.82 (0.64–1.05)	0.113
Model 2	1.76 (1.31–2.36)	< 0.001	1.31 (0.98–1.75)	0.070	1.44 (1.02–2.05)	0.041	0.96 (0.73–1.25)	0.741
Model 3	1.68 (1.24–2.27)	0.001	1.22 (0.91–1.63)	0.193	1.28 (0.89–1.84)	0.178	0.94 (0.72–1.23)	0.639
Model 4	1.68 (1.24–2.28)	0.001	1.22 (0.91–1.64)	0.190	1.22 (0.84–1.77)	0.291	0.91 (0.69–1.19)	0.485
Model 5	1.66 (1.22–2.26)	0.001	1.23 (0.91–1.66)	0.180	1.26 (0.86–1.84)	0.229	0.91 (0.69–1.20)	0.495

Model 1 = unadjusted, Model 2 = adjusted for age and sex, Model 3 = Model 2 + BMI and diabetes duration, Model 4 = Model 3 + systolic blood pressure, HbA1c, and eGFR (MDRD), Model 5 = Model 4 + use of statins, diabetes medication, alcohol consumption, and smoking status.

*PAID-K* Korean version of the Problem Areas in Diabetes Survey.

Among 1862 participants, 928 patients (49.8%) completed 3 years of follow-up after the survey. Changes in HbA1c levels during the 3-year follow-up after the survey were analyzed (Supplementary Fig. 2). At baseline, individuals with high distress had higher HbA1c levels than those without high distress. The difference between the groups persisted for 3 years of follow-up (Supplementary Fig. 2a). The proportion of patients with well-controlled diabetes, defined as HbA1c < 6.5%, was significantly lower in the high distress group at baseline and after 3 years of follow-up (High distress group: 19.4% (68/350) and 17.7% (62/350), Low distress group: 29.4% (170/578) and 28.7% (166/578), baseline and after 3 years, respectively) (Supplementary Fig. 2b). Among the 928 patients, the individuals with HbA1c < 6.5% at the time of survey (n = 238) were separately analyzed (Supplementary Fig. 2c,d). The HbA1c levels at baseline did not differ between the groups according to the presence of high distress (Supplementary Fig. 2c). However, after 3 years of follow-up, only 41.2% (28 of 68) of patients in the high distress group maintained a HbA1c level < 6.5%, whereas 60.6% (103 of 170) of patients in the low distress group maintained well controlled diabetes (p = 0.007, Supplementary Fig. 2d). An additional separate analysis was performed on individuals with a baseline HbA1c ≥ 6.5% (n = 690) at the time of the survey (Supplementary Fig. 2e,f). Over the course of the 3-year follow-up period, the high distress group consistently maintained significantly elevated HbA1c levels (all p < 0.05) (Supplementary Fig. 2e). However, after the 3-year follow-up, there was no significant difference in the proportion of patients achieving an HbA1c level < 6.5% (15.4% vs. 12.1%, p = 0.209) (Supplementary Fig. 2f).

The risk of uncontrolled diabetes, defined as HbA1c ≥ 6.5% at 3 years after the survey, was evaluated. In the univariable analysis, a high PAID-K score (≥ 40) was associated with poor glycemic control at 3 years after the survey (Supplementary Table 1). However, this association was no longer significant after adjusting for variables that were significant in the univariable analysis. When we further analyzed the subgroup of patients with HbA1c < 6.5% at baseline, a high (≥ 40) PAID-K score was associated with poor glycemic control at 3 years after the survey, and this remained significant after adjusting for multiple variables. Other factors such as young age, long diabetes duration, high baseline HbA1c, and insulin treatment were associated with uncontrolled diabetes at 3 years after the survey. When we additionally analyzed the subgroup of patients with HbA1c ≥ 6.5% at baseline, only young age and high baseline HbA1c were associated with uncontrolled diabetes at 3 years after the survey.

Given the potential association between diabetic neuropathy and emotional distress^29^, we conducted a subgroup analysis based on the presence of diabetic neuropathy. The baseline characteristics of the participants, stratified according to the presence of diabetic neuropathy, are shown in Supplementary Table 2. In patients with diabetic neuropathy, a high PAID-K score was associated with young age, long diabetes duration, high HbA1c, high carbohydrate intake and low fiber intake. However, in patients without diabetic neuropathy, the association with other factors was attenuated, and solely age and diabetes duration exhibited significant associations with the PAID-K score. (Supplementary Table 3).

## Discussion

In this study, patients with high diabetes distress had higher glucose levels at baseline, and their glycemic control was poorly maintained. In addition, high diabetes distress was associated with higher odds of having diabetic neuropathy. A higher PAID-K score was associated with younger age, female gender, longer duration of diabetes, and increased carbohydrate intake.

The causal relationship between various individual/metabolic factors and diabetic distress has not been clearly elucidated. The level of diabetes distress was generally high in patients who are women, younger, non-white, those with high BMI, and those treated with insulin^30^. Fisher et al. reported that female sex, young age, depressive disorder, poor diet, and low exercise were risk factors for future diabetes distress^31^. Similar to previous studies, we observed that high PAID-K score was associated with younger age, longer duration of diabetes, and higher carbohydrate intake. Although the mechanism is uncertain, HbA1c level and dietary intake were not associated with PAID-K score in patients without diabetic neuropathy.

Despite the earlier hypothesis that diabetes distress may improve glucose control in patients with diabetes, the majority of previous studies have found that diabetes distress is actually linked to higher HbA1c levels and even negative metabolic outcomes^1^. The poor glycemic control observed in our patients with high distress could partially be explained by their lower levels of self-care, represented by unhealthier dietary patterns. Although food choices in response to stress may differ according to age, ethnicity, and cultural background, diabetes distress may affect food choices^32^. Martyn-Nemeth et al. also reported that diabetes distress was associated with overeating, leading to poor glucose control^33^. Additional factors such as younger age, long diabetes duration, high HbA1c levels, and insulin treatment were identified as significant contributors to uncontrolled diabetes after a 3-year follow-up. Young age populations often show poor glycemic control^34^, and prolonged DM duration is associated with decreased insulin secretion function^35^, potentially leading to uncontrolled glucose level. Insulin treatment may reflect reduced insulin secretion capacity and is frequently employed as a therapeutic strategy for patients with poor glycemic control^36^.

Depression is associated with an increased risk for diabetic neuropathy, retinopathy, nephropathy, sexual dysfunction, and macrovascular complications^37,38^. However, aside emotional stress, such as depression and anxiety, the association between diabetes distress and diabetic complications has not been firmly established yet. In this study, we observed that high distress was associated with diabetic neuropathy after adjusting for multiple confounding factors; however, the underlying mechanism is unclear, and neurochemical hormones may explain the association between diabetes distress and neuropathy. Suffering from the challenges of diabetes, the physiology of the neuroendocrine system and neurotransmitters, such as serotonin and norepinephrine may change and affect development of diabetes distress^39^. Medications that inhibit serotonin and norepinephrine uptake are currently used to treat diabetic neuropathy^40^, suggesting a role for neurochemical hormones in linking diabetes distress and diabetic neuropathy. Our study findings also indicate that even within the high distress group, there could be varying backgrounds and metabolic characteristics based on the coexistence of diabetic neuropathy. This highlights the importance of considering the presence of diabetic neuropathy when evaluating the effect of diabetes distress on patients’ well-being and glycemic control.

In this study, we did not find a significant association between diabetes distress and albuminuria. A recent study by Hayashino et al. also explored the relationship between diabetes distress and the development and progression of diabetic nephropathy^41^. There was no association between diabetes distress and diabetic nephropathy at baseline, which seems consistent with our study. However, they found that diabetes distress was associated with the progression from microalbuminuria to macroalbuminuria in male individuals. Considering the result of the current study, the consistently poor glycemic control within the high distress group could potentially play a role in accelerating the progression of diabetic complications.

This study has several limitations. First, the distress survey was performed only once during the study period; therefore, we could not assess the change in diabetes distress and how the stress change affected glycemic control or the development of complications. Second, only half of the study participants completed the 3-year follow up after the survey. Third, a causal relationship between diabetes distress and complications was not identified due to the study design. Additionally, variables with more than 25% of missing data were excluded from the analysis. Therefore, several factors such as homeostatic model assessment indices and the insulinogenic index were not analyzed. A well-controlled prospective study is needed to clarify the causal relationship and investigate the association between diabetes distress, glycemic control, and complications. Lastly, diabetes distress can directly influence patients' compliance, physical activity, and carbohydrate intake, all of which are interrelated factors. Consequently, even with the inclusion of numerous factors as confounding variables, there might still be limitations in comprehensively accounting for all the complex interactions between these factors. However, this study had several notable strengths. We evaluated diabetes distress and related parameters, including dietary behavior, glycemic control, and diabetic complications, simultaneously. Additionally, this study was conducted in an East Asian population. Although diabetes distress has been shown to vary based on ethnicity and cultural background^10^, there is currently limited research available on diabetes distress among East Asian populations. Only a few studies have reported on diabetes distress and its association with glycemic control and diabetic complications in the Asian population. We believe that this study can raise awareness about diabetes distress and establish a foundation for future research.

In conclusion, high diabetes distress was associated with persistent hyperglycemia and higher odds of having diabetic neuropathy. While establishing a definitive causal relationship is challenging, this emphasizes the necessity for a more proactive approach to measuring diabetes distress and managing poor glycemic control in individuals experiencing diabetic distress, along with a thorough evaluation for diabetic neuropathy.

### Supplementary Information

Supplementary Information.

## Data Availability

The datasets generated during and/or analysed during the current study are available from the corresponding author on reasonable request.
