# Supplementary material for: Impact of diabetes distress on glycemic control and diabetic complications in type 2 diabetes mellitus
Source: Sci Rep. 2024 Mar 6;14:5568. doi: 10.1038/s41598-024-55901-0 (PMC10917807; doi:10.1038/s41598-024-55901-0)
Supplement: Supplementary file 1 — Supplementary Information. [file 41598_2024_55901_MOESM1_ESM.docx]

Supplementary Table 1. Odds ratio of having uncontrolled diabetes (HbA1c ≥6.5%) after three years in the study participants

| Uni | OR | 95% CI | p value | Multi | OR | 95% CI | p value |
| --- | --- | --- | --- | --- | --- | --- | --- |
| Parameters |  |  |  |  |  |  |  |
| All Patients (N=928) | | | | | | | |
| High distress | 1.87 | 1.35–2.57 | <0.001 |  | 1.38 | 0.95–2.02 | 0.094 |
| Age | 0.97 | 0.96–0.99 | 0.001 |  | 0.97 | 0.95–0.99 | 0.001 |
| Female | 1.12 | 0.83–1.50 | 0.467 |  |  |  |  |
| DM duration | 1.09 | 1.06–1.19 | <0.001 |  | 1.05 | 1.02–1.08 | 0.005 |
| BMI | 1.05 | 1.00–1.11 | 0.056 |  |  |  |  |
| HbA1c | 5.13 | 3.84–6.86 | <0.001 |  | 4.37 | 3.24–5.89 | <0.001 |
| LDL-cholesterol | 1.00 | 0.99–1.00 | 0.438 |  |  |  |  |
| eGFR | 1.00 | 0.99–1.00 | 0.245 |  |  |  |  |
| Insulin treatment | 2.84 | 1.65–4.88 | <0.001 |  | 1.33 | 0.70–2.53 | 0.378 |
| smoking (current) | 0.89 | 0.60–1.31 | 0.549 |  |  |  |  |
| alcohol (current) | 0.90 | 0.66–1.24 | 0.530 |  |  |  |  |
| Calorie intake, daily | 1.00 | 1.00–1.00 | 0.820 |  |  |  |  |
| Patients with well controlled (HbA1c <6.5%) diabetes at survey (N=238) | | | | | | | |
| High distress | 2.20 | 1.24–3.89 | 0.007 |  | 2.01 | 1.03–3.91 | 0.041 |
| Age | 0.98 | 0.95–1.01 | 0.108 |  | 0.95 | 0.92–0.98 | 0.002 |
| Female | 0.81 | 0.48–1.37 | 0.435 |  |  |  |  |
| DM duration | 1.08 | 1.03–1.14 | 0.002 |  | 1.09 | 1.02–1.16 | 0.007 |
| BMI | 1.07 | 0.98–1.17 | 0.149 |  |  |  |  |
| HbA1c | 26.10 | 7.64–89.10 | <0.001 |  | 30.67 | 8.30–113.25 | <0.001 |
| LDL-C | 1.00 | 0.99–1.01 | 0.677 |  |  |  |  |
| eGFR | 1.00 | 0.99–1.01 | 0.538 |  |  |  |  |
| Insulin treatment | 3.30 | 1.23–8.83 | 0.018 |  | 3.25 | 1.03–10.31 | 0.045 |
| smoking (current) | 0.87 | 0.43–1.73 | 0.684 |  |  |  |  |
| alcohol (current) | 1.23 | 0.71–2.15 | 0.466 |  |  |  |  |
| Calorie intake, daily | 1.00 | 1.00–1.00 | 0.365 |  |  |  |  |
| Patients with poorly controlled (HbA1c ≥6.5%) diabetes at survey (N=690) | | | | | | | |
| High distress | 1.33 | 0.85–2.08 | 0.210 |  | 1.13 | 0.71-1.82 | 0.603 |
| Age | 0.98 | 0.95–1.00 | 0.055 |  | 0.97 | 0.95–1.00^*^ | 0.039 |
| Female | 1.17 | 0.76–1.80 | 0.479 |  |  |  |  |
| DM duration | 1.05 | 1.01–1.09 | 0.014 |  | 1.04 | 1.00-1.08 | 0.064 |
| BMI | 1.05 | 0.97–1.13 | 0.253 |  |  |  |  |
| HbA1c | 2.72 | 1.83-4.05 | <0.001 |  | 2.53 | 1.68-3.80 | <0.001 |
| LDL-C | 1.00 | 1.00–1.01 | 0.397 |  |  |  |  |
| eGFR | 1.00 | 0.99–1.01 | 0.668 |  |  |  |  |
| Insulin treatment | 1.78 | 0.89-3.55 | 0.101 |  | 1.02 | 0.48-2.17 | 0.959 |
| smoking (current) | 0.93 | 0.53-1.64 | 0.811 |  |  |  |  |
| alcohol (current) | 0.79 | 0.50–1.25 | 0.312 |  |  |  |  |
| Calorie intake, daily | 1.00 | 1.00–1.00 | 0.779 |  |  |  |  |

A score ≥40 on the Korean version of the Problem Areas in Diabetes Survey was defined as high distress.

OR, odds ratio; CI, confidence interval; DM, diabetes mellitus; BMI, body mass index; HbA1c, glycated hemoglobin; LDL, low-density lipoprotein; eGFR, estimated glomerular filtration rate.

^*^0.995 before rounding

Supplementary Table 2. Baseline characteristics of study participants according to the presence of diabetic neuropathy

| Variables | Without DN | With DN | p value |
| --- | --- | --- | --- |
|  | (N=920) | (N=294) |  |
| Female, n (%) | 409 (44.5) | 139 (47.3) | 0.397 |
| Age, years | 58.0 (52.0-64.0) | 60.0 (53.0-67.0) | 0.003 |
| Duration of diabetes, years | 8.0 (4.0-13.0) | 10.0 (6.0-16.0) | <0.001 |
| BMI, kg/m^2^ | 24.8 (22.8-26.6) | 25.6 (23.7-27.7) | <0.001 |
| Waist circumference, cm | 86.0 (81.5-91.0) | 88.0 (84.0-94.0) | <0.001 |
| Systolic BP, mmHg | 125.0 (117.0-132.0) | 125.0 (118.0-131.0) | 0.797 |
| Diastolic BP, mmHg | 78.0 (70.0-81.0) | 78.0 (70.0-80.0) | 0.420 |
| Fasting blood glucose, mg/dL | 129.0 (111.0-148.0) | 132.0 (113.5-153.0) | 0.150 |
| HbA1c, % | 7.0 (6.5-7.7) | 7.2 (6.5-8.1) | 0.040 |
| Triglyceride, mg/dL | 107.5 (74.3-160.8) | 125.0 (84.0-177.8) | <0.001 |
| HDL-cholesterol, mg/dL | 49.0 (42.0-58.0) | 48.0 (40.0-56.0) | 0.023 |
| LDL-cholesterol, mg/dL | 82.2 (62.6-102.1) | 85.2 (64.5-107.1) | 0.149 |
| uACR | 9.8 (5.6-27.3) | 14.4 (7.1-56.2) | <0.001 |
| eGFR MDRD, mL/min/1.73 m^2^ | 90.6 (76.4-109.4) | 80.5 (67.4-95.9) | <0.001 |
|  |  |  |  |
| PAID-K score | 31.3 (16.3-43.8) | 37.5 (23.8-51.3) | <0.001 |
| Presence of high distress, n(%) | 298 (32.4) | 135 (45.9) | <0.001 |

A score ≥40 on the Korean version of the Problem Areas in Diabetes Survey was defined as high distress. Continuous variables are expressed as median with interquartile range. Mann-Whitney U were used for continuous variables analysis.

DN, diabetic neuropathy; BMI, body mass index; BP, blood pressure; HbA1c, glycated hemoglobin; HDL, high-density lipoprotein; LDL, low-density lipoprotein; uACR, urinary albumin-creatinine ratio; eGFR, estimated glomerular filtration rate; MDRD, modification of diet in renal disease; PAID-K, Korean version of the Problem Areas in Diabetes Survey.

Supplementary Table 3. Association of PAID-K score with participants’ clinical characteristics and laboratory findings in multiple linear regression model according to the presence of diabetic neuroapthy

|  | PAID-K score | |
| --- | --- | --- |
| Parameters | Standardized coefficients (β) | *p*-value |
| In patients with diabetic neuropathy |  |  |
| Age, years | -0.126 | 0.001 |
| Female | 0.079 | 0.053 |
| Diabetes duration, years | 0.105 | 0.006 |
| HbA1c, % | 0.090 | 0.012 |
| HDL-cholesterol, mg/dL | 0.010 | 0.780 |
| LDL- cholesterol, mg/dL | 0.055 | 0.114 |
| Energy intake, kcal/day | -0.005 | 0.915 |
| Carbohydrate intake, % energy | 0.084 | 0.026 |
| Fiber intake, g/1,000 kcal | -0.114 | 0.005 |
| In patients without diabetic neuropathy |  |  |
| Age, years | -0.152 | 0.030 |
| Female, years | 0.116 | 0.131 |
| Diabetes duration, years | 0.160 | 0.021 |
| HbA1c, % | -0.072 | 0.271 |
| HDL-cholesterol, mg/dL | 0.041 | 0.536 |
| LDL- cholesterol, mg/dL | 0.083 | 0.202 |
| Energy intake, kcal/day | -0.025 | 0.780 |
| Carbohydrate intake, % energy | -0.041 | 0.560 |
| Fiber intake, g/1,000 kcal | 0.028 | 0.709 |

PAID-K, Korean version of the Problem Areas in Diabetes Survey; HbA1c, glycated hemoglobin; HOMA-IR, homeostatic model assessment for insulin resistance; HDL, high-density lipoprotein; LDL, low-density lipoprotein.

Supplementary figure 1. Study flow


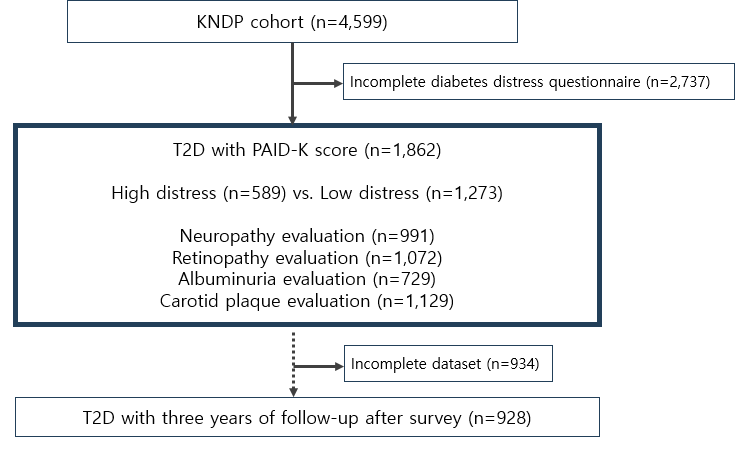


KNDP, Korean National Diabetes Program; T2D, type 2 diabetes; PAID-K, Korean version of the Problem Areas in Diabetes Survey.

Supplementary Figure 2. Glycemic control in study participants at baseline and after three years


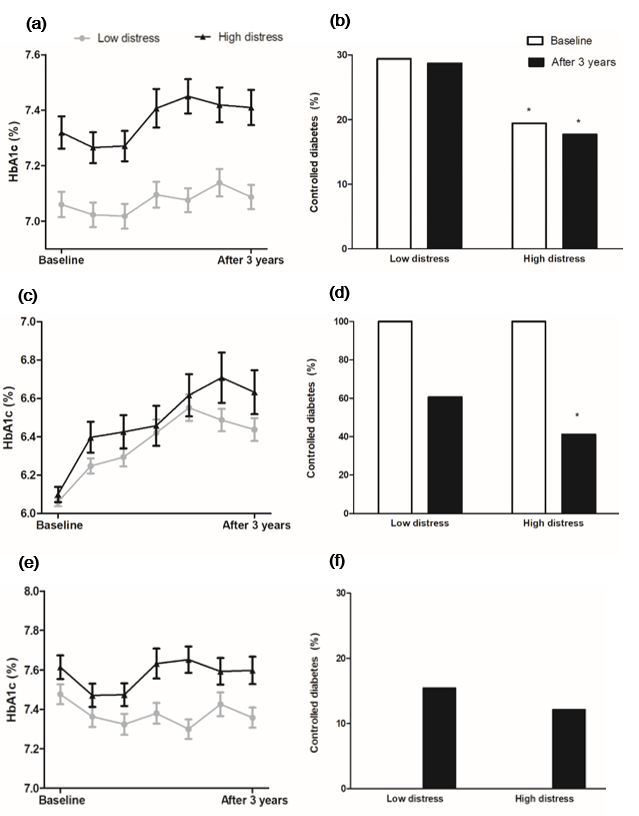


a) HbA1c changes in total study participants

b) Percentage of study participants achieving HbA1c < 6.5%

c) HbA1c changes in study participants whose HbA1c were < 6.5% at baseline

d) Percentage of study participants achieving HbA1c < 6.5% among those whose baseline HbA1c was < 6.5%

(e) HbA1c changes in study participants whose HbA1c were ≥ 6.5% at baseline

(f) Percentage of study participants achieving HbA1c < 6.5% among those whose baseline HbA1c was ≥ 6.5%

*p < 0.05 vs. low distress group, compared by χ2 analysis
